# Genetic Characterization of Animal* Brucella* Isolates from Northwest Region in China

**DOI:** 10.1155/2018/2186027

**Published:** 2018-05-15

**Authors:** Xiaoan Cao, Youjun Shang, Yongsheng Liu, Zhaocai Li, Zhizhong Jing

**Affiliations:** State Key Laboratory of Veterinary Etiological Biology, Lanzhou Veterinary Research Institute, Chinese Academy of Agricultural Sciences, Lanzhou 730046, China

## Abstract

Animal brucellosis is a reemerging disease in China, particular in northwest China. The* Brucella* species (even genus) are highly conserved; therefore the use of Multilocus sequencing typing (MLST: based on conserved housekeeping loci) is more suitable for discrimination at species or biovar level on* Brucella*. In this study, MLST was used to analyze the characterization of* Brucella* from sheep and yaks during 2015 and 2016. All 66 isolates were collected from northwest China, including Inner Mongolia, Xinjiang, Qinghai, and Gansu provinces. Isolates were cultured on* Brucella* agar medium and identified by MLST. MLST identified five ST types: ST8 (*n* = 55), ST7 (*n* = 2), ST3 (*n* = 5), ST1 (*n* = 2), and ST14 (*n* = 2). This analysis revealed that* B. melitensis* isolates exhibited high single genotypes (ST8) in the most northwest China. MLST of isolates provides helpful information on understanding genetic characterization of* Brucella* in northwest China.

## 1. Introduction

Brucellosis, caused by* Brucella spp*., is a common zoonotic disease worldwide [1]. It has been considered as a reemerging infectious disease in China because of the increasing incidences in the past several years [2].* Brucella melitensis *is responsible for the major causative agent of brucellosis in sheep and human. Sheep and goats are major herbivores in northwest China and are primarily kept by poor rural farmers in pastoral areas [3, 4]. Therefore, brucellosis has an important zoonosis in northwest China.

Multilocus sequencing typing (MLST), as a genotyping tool for assessing genetic diversity and relationships, was widely used to identify and analyze diversity of bacteria and epidemiology characterization [5–8]. Although the data of MLST of* Brucella* stranis in China can not adequately reflect its epidemiological characteristics and relationship between disease emerging and development, the prevalence of genotypes of* Brucella* strains from Inner Mongolia has changed over time in the three stages [9]. The aims of the present study were to identify genotypes of* Brucella* in northwest China and we tried to analyze relationship between disease prevalence and genotypic diversity. Therefore, MLST was used to analyze 66* Brucella* isolates from sheep and yaks during 2015 and 2016.

## 2. Materials and Methods

### 2.1. *Brucella* Strains and DNA Extraction

In the previous study [10], sixty-six isolates were collected from northwest China; 13, 26, 17, and 10 were from Gansu, Inner Mongolia, Xinjiang, and Qinghai provinces, respectively (Table 1).* Brucella* were reproduced in BBL™* Brucella* Broth (BD, USA) with 5% horse serum at 37°C. The 50 ml mid-log phase culture was harvested by centrifugation at 10,000 ×g for 5 min and resuspended in 10 ml PBS (0.01 M, pH 7.2). Total genomic DNA was extracted using a DNeasy Blood & Tissue Kit (Qiagen, Germany) according to the manufacturer's instructions. DNA extracted from all isolates was stored at −20°C.

### 2.2. MLST Genotyping

MLST genotyping was performed by analyzing nine distinct genomic loci, including seven housekeeping genes (gap, aroA, glk, dnaK, gyrB, trpE, and cobQ), one outer membrane protein (omp25), and one intergenic fragment (int-hyp) [11]. PCR amplification was performed as described previously [9]. Sequences obtained from purified PCR products were aligned using MEGA 5 according to published MLST sequences in GenBank (accession numbers AM694191–AM695630) [11]. A local comparison database was established after downloading of relevant data, and distinct alleles identified at the nine selected loci were each given a numerical designation according to the sequences of the defined alleles. Each sequence type over all loci (ST) was predicted by comparisons and analyses based on a local comparison database established using MEGA 5 and a web-based MLST service (*Brucella* Base, https://pubmlst.org/brucella/). DNA preparations from the* B. melitensis* 16 M,* B. abortus* 544, and* B. suis* 1330 reference strains were used as controls.

### 2.3. Analysis of MLST DATA

To clarify the molecular characteristics and evolutionary relationships of brucellosis in northwest China, all 66 isolates were analyzed using BioNumerics version 7.6. Using the same software, clustering analysis was performed using minimal spanning tree [12, 13]. The resulting genotypes were compared using the web-based MLST database (https://pubmlst.org/brucella/). Genotypic diversification of* Brucella* in northwest China was analyzed using this study and published data [9, 12–14].

## 3. Results

### 3.1. MLST Results

**Figure 1:**
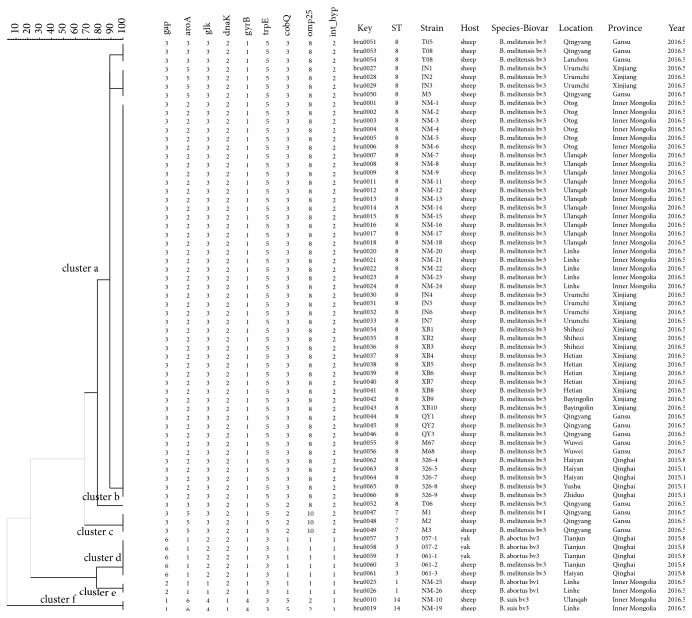
Dendrogram based on the MLST genotyping assay showing relationships of 66* Brucella* isolates. Key: serial number for the 66 isolates; ST: MLST genotype fo isolates; strain: the number conferred to isolates; host: the hosts from which the bacteria were isolated; location: sample specific location (Table 1 in detail); province: the regions of sample collection; year: the time of isolation of bacteria.

MLST analysis showed that five known MLST genotypes were identified: ST3 (6-1-2-2-1-3-1-1-1; *n* = 5), ST1 (2-1-1-2-1-3-1-1-1; *n* = 2), ST8 (3-3-3-2-1-5-3-8-2; *n* = 44), ST7 (3-5-3-2-1-5-3-8-2; *n* = 3), and ST14 (1-6-4-1-4-3-5-2-1; *n* = 2). Clustering analysis by using BioMumerics software showed that the 66 isolates formed six main clusters (a–f).* B. melitensis *was distributed in cluster a–c, and genotype ST8 plays a dominant role in isolates (53/66). Genotypes ST1 belonged to* B. abortus*; genotype ST14 belonged to* B. suis*; genotypes ST7 and ST8 belonged to* B. melitensis*, while 5 isolates identify as ST3, including two* B. melitensis *strains and three* B. abortus* strains, which were isolated from Tianjun county, Qinghai province (Figure 1). Therefore, MLST is more suitable for discrimination at species or biovar level on* Brucella* species. Analysis of MLST was conducted in 69* Brucella* strains involving nucleotide sequences of 4396 positions. The result showed that 28 segregating sites were presented among those loci.

### 3.2. Molecular Epidemiology of* Brucella* in Northwest China

Among 66 isolates,* B. melitensis* isolates were identified as genotypes ST3, ST7, and ST8;* B. abortus* isolates were ST1 and ST3; and* B. suis* isolates were ST14. ST8 was a dominant genotype in those* B. melitensis* isolates, and it is a widespread* Brucella* genotype in northwest China. The results also showed that* B. abortus *infected sheep, but worryingly,* B. suis *biovar 3 with genotype ST14 emerged in sheep in Inner Mongolia. On the other hand, two isolates from sheep in Qinghai province were* B. melitensis* biovar 3, while MLST result presented genotype ST3, which was identified from isolates in yaks.

## 4. Discussion

In the present study, we used MLST methods to genotype* Brucella* isolates from northwest China. A total of 66* Brucella* isolates were examined by MLST typing. In the previous study, all isolates were* B. melitensis*,* B. abortus, *and* B. suis*.* B. melitensis* was dominant epidemic strain in animal and* B. abortus *and* B. suis *also infected sheep;* B. suis *biovar 3 especially emerged in Inner Mongolia.* B. melitensis *genotype ST8 not only was the predominant genotype in sheep but also responded for human brucellosis. These results reveal that human brucellosis in northwest China is closely related to infectious sheep.

In the south of Gansu province,* B. melitensis* isolates have two genotypes (ST7 and ST8). It was indicated that the epidemiology of* Brucella* in there was diversified. MLVA analysis of China isolates suggested that there were three predominant but different lines of* Brucella* transmission in China, while a common thing is that the transmission lines are from north to south [15], but MLST analysis has a comprehensive understanding of* Brucella* epidemic in China. So far, one of the regrets is that there is little data related to genotype study involved* Brucella* in the central and western regions in China. Therefore, change and transmission line of brucellosis are unclear from the south of Gansu to the central and western regions in China, and the causes and consequence of* Brucella* diversity should be investigated by molecular epidemiology analysis of emerging* Brucella* in the central and western regions in China.

**Figure 2:**
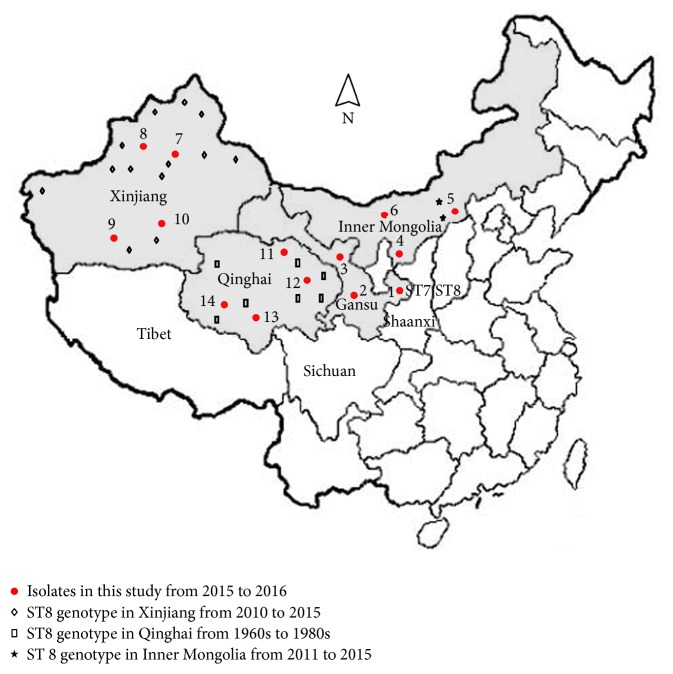
Geographic distribution of* Brucella* genotype ST8 in northwest China. Dotted (red) countries: sampling area (Table 1 in detail); other shapes: the other data collected in different provinces in northwest China from [12–14].

Collection of MLST data of isolates from northwest China: ST8 was a dominant genotype of* B. melitensis* which were responsible for sheep and human brucellosis (Figure 2) [9, 13, 14]. MLST analysis of* Brucella* strains isolated from the 1980s in Qinghai province revealed that genotype ST8 of* B. melitensis* was widely spread in sheep, blue sheep, and human [13].* B. melitensis* ST8 genotype was also identified from isolates in sheep and human in Gansu, Xinjiang, and Inner Mongolia [12, 14].

In the previous study, brucellosis in China was divided into three periods, high incidence (1950–1960s), decline (1970–1980s), and reemergence (1990–2000s) [9]. On the other hand, brucellosis has been rising every year since the beginning of the 21st century, especially outbreaks in parts of the northwest China in recent years [2, 16]. In Inner Mongolia, 61.11% (11/18), 14.29% (3/21), 47.62% (10/21), and 100% (116/116) genotype ST8* Brucella* were isolated in 1950–1960s, 1970–1980s, 1990–2000s, and 2010–2015 stages, respectively [9, 12]. Those results revealed that genotype ST8* B. melitensis *is an important epidemiological marker for trend of brucellosis of epidemics in northwest China. It is further suggested that the ST8 is an extensive endemic in northwest region of China. Conversely, lower genetic diversity and crowding effects may favor transmission and select faster replicating organisms with major zoonotic potential [16–18], which will increase threatening of brucellosis for animals and human. Recently, a research involving MLST analysis of* Brucella* isolates in China between 1953 and 2013 showed that a total of 206 isolates have been identified as 32 MLST genotypes (STs), which included 13 new STs (ST71-83), although ST8 was a dominant genotype [19]. It is implied that the study of* Brucella* genotype in larger scale epidemic strains should be carried out in order to understand the characteristics of* Brucella* epidemic and genetic variation.

## 5. Conclusion

MLST was used to analyze 66* Brucella* isolates from the northwest China. 83% (55/66) were genotype ST8. Analysis of genotype of* Brucella* strains from the northwest China further confirmed that ST8 was a dominant* Brucella* genotype responding for sheep and human disease.

## Figures and Tables

**Table 1:** Geographical distribution and genotype of *Brucella* isolates in northwest China.

Province	Host	Locality ID	Region	Species	Genotype	Isolate size
Gansu	Sheep	1	Qingyang	*B. melitensis*	ST7	4
					ST8	6
		2	Lanzhou	*B. melitensis*	ST8	1
		3	Wuwei	*B. melitensis*	ST8	2
Inner Mongolia	Sheep	4	Otog	*B. melitensis*	ST8	6
		5	Ulanqab	*B. suis*	ST14	1
				*B. melitensis*	ST8	11
		6	Linhe	*B. abortus*	ST1	2
				*B. suis*	ST14	1
				*B. melitensis*	ST8	5
Xinjiang	Sheep	7	Urumchi	*B. melitensis*	ST8	7
		8	Shihezi	*B. melitensis*	ST8	3
		9	Hetian	*B. melitensis*	ST8	5
		10	Bayingolin	*B. melitensis*	ST8	2
Qinghai	Yaks	11	Tianjun	*B. abortus*	ST3	3
	Sheep			*B. melitensis*	ST3	1
		12	Haiyan	*B. melitensis*	ST3	1
					ST8	3
		13	Yushu	*B. melitensis*	ST8	1
		14	Zhiduo	*B. melitensis*	ST8	1

Province: region of sample collection; host: host from which the bacteria were isolated; locality ID corresponds to the counties of isolation; region: region from which the samples and isolates were collected.
